# Supplementary material for: Neuronal cell-subtype specificity of neural synchronization in mouse primary visual cortex
Source: Nat Commun. 2019 Jun 10;10:2533. doi: 10.1038/s41467-019-10498-1 (PMC6557841; doi:10.1038/s41467-019-10498-1)
Supplement: Supplementary file 1 — Supplementary Information [file 41467_2019_10498_MOESM1_ESM.pdf]

Physiologically distinct subtypes of Somatostatin expressing inhibitory interneurons in mouse primary visual cortex.

Ulf Knoblich, Lawrence Huang, Hongkui Zeng, Lu Li

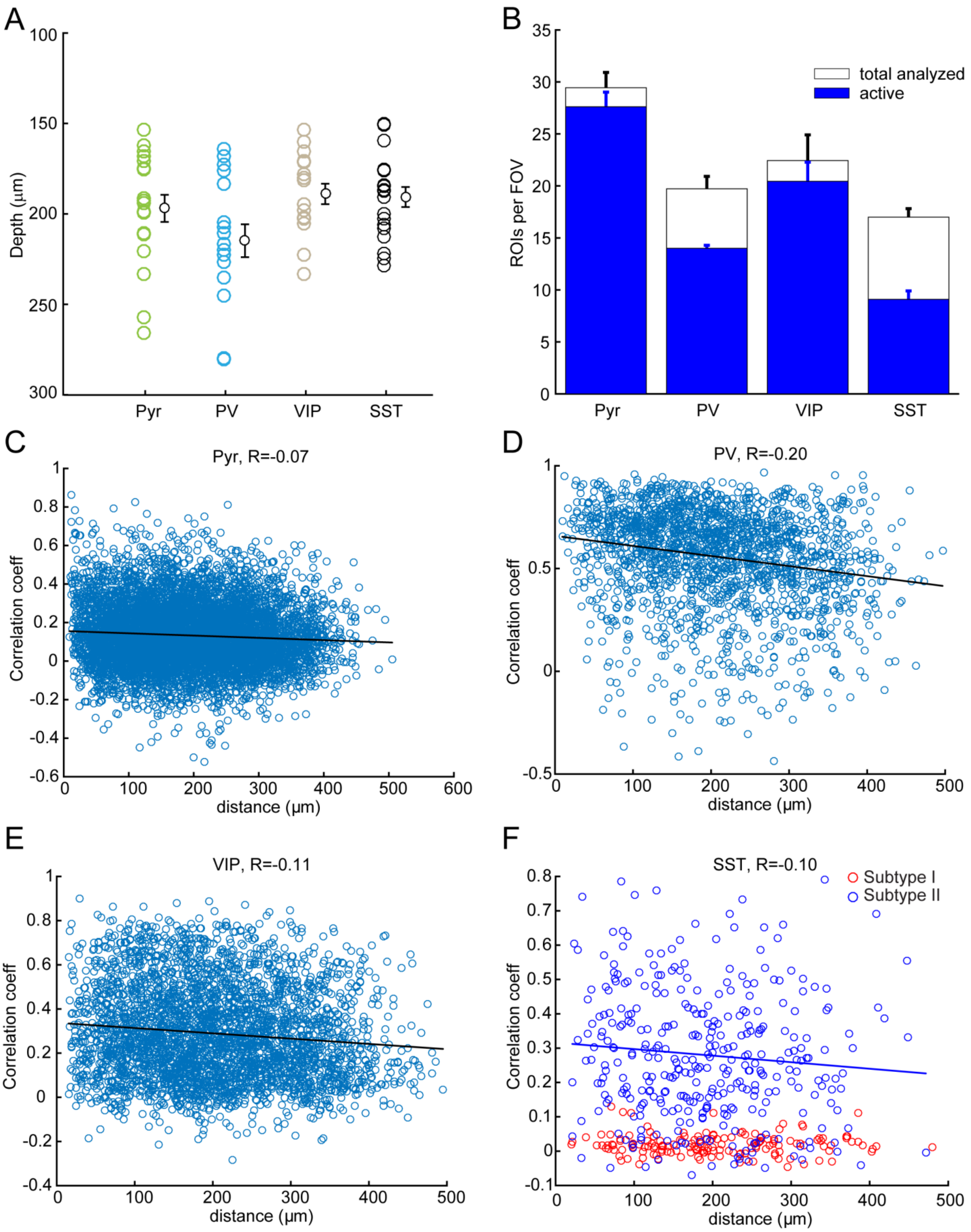
A
Depth (µm)
100
150
200
250
300
Pyr
PV
VIP
SST
B
ROIs per FOV
total analyzed
active
C
Pyr, R=-0.07
Correlation coeff
distance (µm)
D
PV, R=-0.20
E
VIP, R=-0.11
F
SST, R=-0.10
Subtype I
Subtype II

Supplementary Figure 1. A. Distributions of Ca imaging depths of Pyr (green), PV (light blue), VIP (gray) and SST (black) neurons, respectively. All FOVs were imaged around 200 μm underneath the pial surface. Only analyzed FOVs were shown. **B.** Average numbers of total ROIs (white) and fluorescently active ROIs (blue) per FOV in Pyr, PV, VIP and SST neurons, respectively. A considerable portion of SST neurons (46%) were fluorescently inactive. **C – F**. Relationship between correlation strength and spatial distance between pairs of active Pyr (**C**), PV (**D**), VIP (**E**) and SST (**F**) neurons, respectively. Pyr neurons, on average, showed a weaker correlation compared with INs. PV INs, on the other hand, were homogeneously and most strongly correlated, even in awake brain. VIP INs were in between Pyr and PV neurons. For SST INs, Subtype I and II were plotted separately. Difference between neuronal cell-types was revealed by binning as shown in Fig. 2I, although overall the correlation strength reduced along distance.

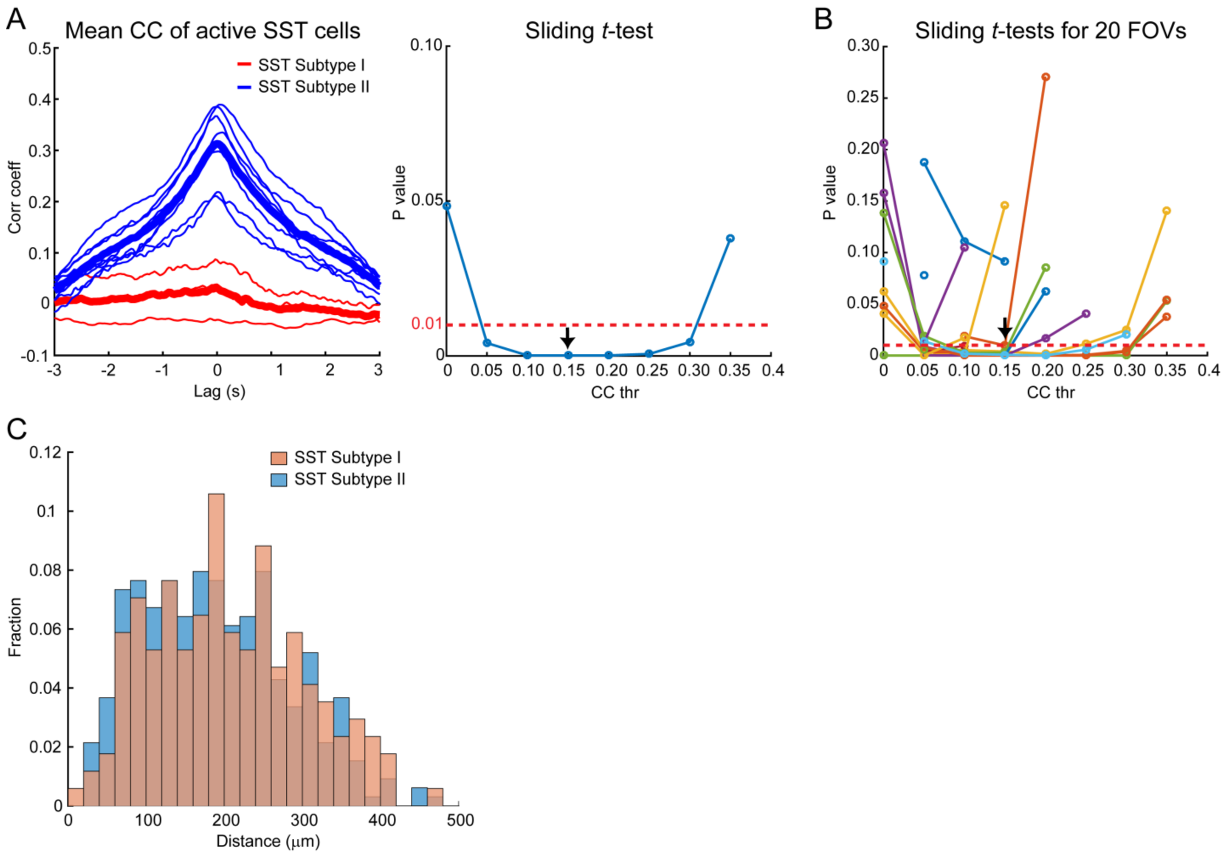

Supplementary Figure 2. A. Left: Mean CC curves for active ROIs in the FOV shown in Fig. 1G. Each curve represented the data of an individual ROI averaged across its all active neighbors. Right: Separation between SST Subtype I and II. A sliding *t*-test was conducted on the distribution of mean CCs of active SST ROIs at zero-time lag to determine the statistical significance. Within this FOV, Subtype I and II were well separated, resulting in a wide range of possible CC threshold. **B.** Sliding *t*-tests for all (n = 20) FOVs in SST animals. Overall the separation between Subtype I and II was significant ($p < 0.01$) for the majority of FOVs (70%, 14 of 20). Thus the mid-point value of 0.15, as shown by the population data, was used as the empirical threshold for all cell types. **C.** Histograms of between-neuron distance of SST Subtype I neurons (orange), overlaid with that of Subtype II (light blue). SST Subtype I neurons were more evenly distributed spatially within the x-y plane, compared with Subtype II. SST Subtype II exhibited a leftward shift, especially within 200 μm, compared with that of SST Subtype I.

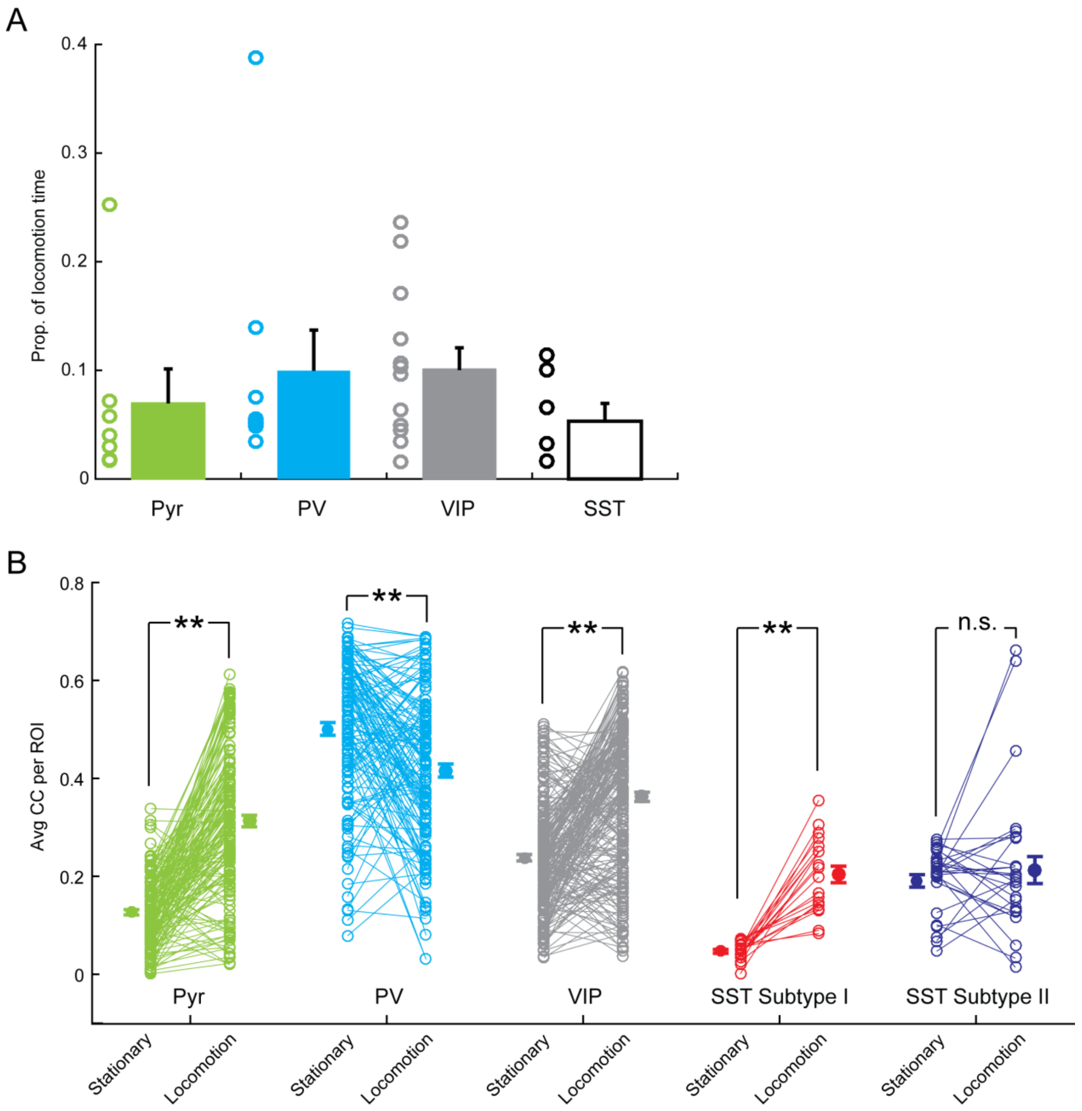

Supplementary Figure 3. Locomotion selectively increased within-type CC in SST Subtype I neurons. **A.** Proportion of locomotion time in a subset of imaging experiments in which mice continuously ran for > 2 sec. Pyr (mean ± s.e.m, same below): 0.07 ± 0.03, from 7 FOVs in 3 mice; PV: 0.10 ± 0.04, from 9 FOVs in 3 mice; VIP: 0.08 ± 0.01, from 13 FOVs in 3 mice; SST: 0.05 ± 0.02, from 6 FOVs in 2 mice. **B.** Within-type CC monotonically increased in SST Subtype I neurons during locomotion, but all other (sub)type neurons showed bidirectional changes. CC between active neuron pairs were averaged for each cell, and compared between stationary and locomotion states. Pyr: 0.13 ± 0.005 vs. 0.31 ± 0.01, n = 162 ROIs, $p < 0.001$, *t*-test; PV: 0.50 ± 0.01 vs. 0.42 ± 0.01, n = 141 ROIs, $p < 0.001$, *t*-test; VIP: 0.24 ± 0.007 vs. 0.36 ± 0.009, n = 251 ROIs, $p < 0.001$, *t*-test.

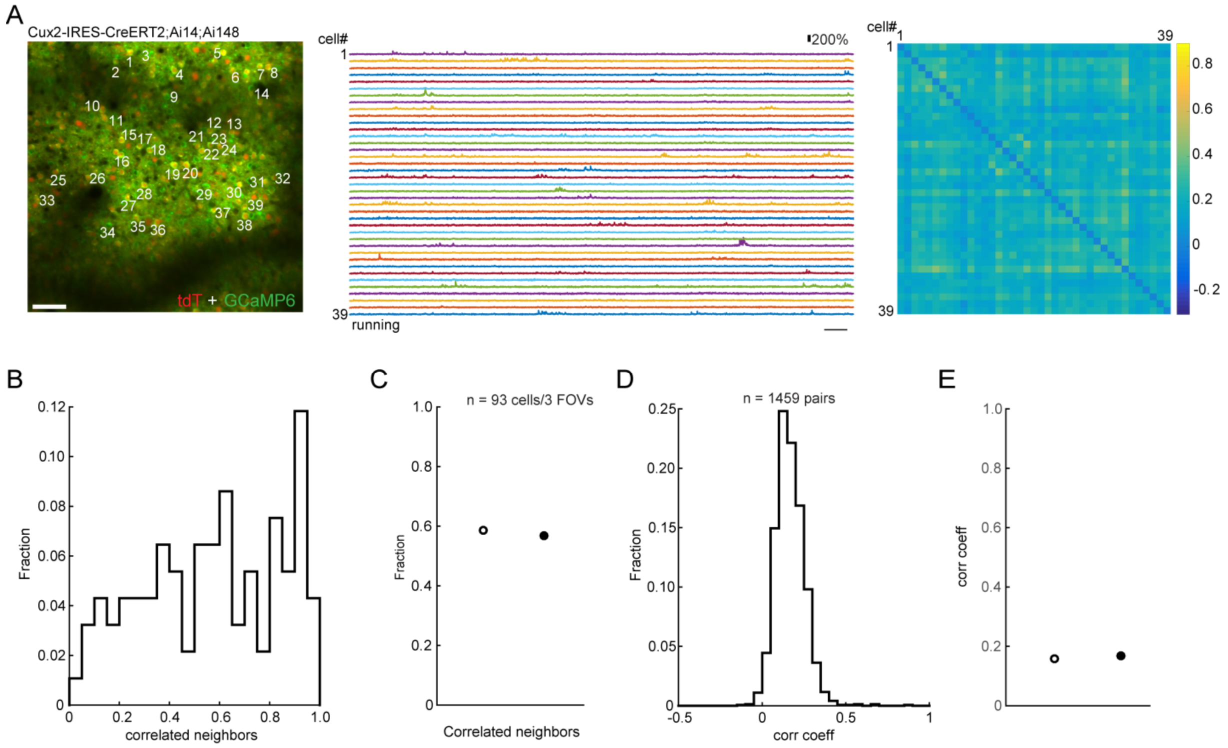

Supplementary Figure 4. Anesthesia reduced the activity level of Pyr neurons but enhanced the pair-wise correlation. **A.** Example Ca imaging in Isoflurane anesthetized Cux2-CreERT2;Ai14;Ai148 (GCaMP6f) mice (n = 1). Figure panels were labeled as in Fig. 1D. **B – D.** Correlativity of V1 L2/3 Pyr neurons, measured by within-type, pair-wise spontaneous CC, was fairly strong under anesthesia (n = 93 of 127 active Pyr neurons of 3 FOVs). **B.** Population histograms showing the within-type correlativity in Pyr neurons under Isoflurane anesthesia. Note the rightward shift toward strong correlation compared with awake animals. **C.** Median (0.59, empty circle) and mean (0.57, solid) values of the fraction of correlated neighbor. **D.** CC distributions of Pyr (n = 1459 pairs) under Isoflurane anesthesia. **E.** Median (0.16, empty circle) and mean (0.17, solid) values of CC. All these data confirmed V1 cortical Pyr network was under a ‘synchronised’ mode under anesthesia.

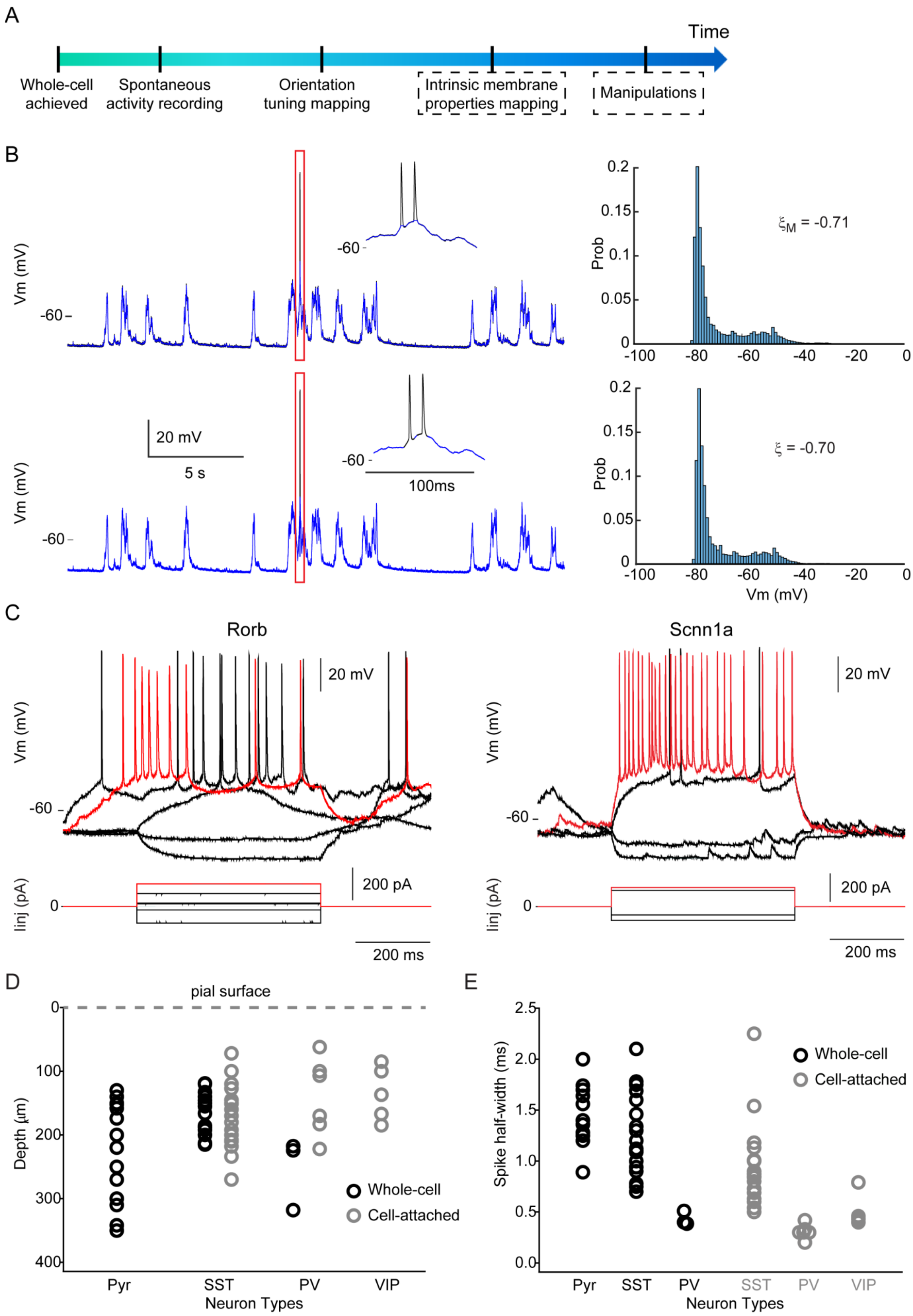

A
Time
Whole-cell achieved
Spontaneous activity recording
Orientation tuning mapping
Intrinsic membrane properties mapping
Manipulations
B
Vm (mV)
-60
20 mV
5 s
100ms
Prob
ξM = -0.71
ξ = -0.70
Vm (mV)
C
Rorb
Scnn1a
20 mV
200 pA
200 ms
Iinj (pA)
D
pial surface
Depth (µm)
Whole-cell
Cell-attached
Pyr
SST
PV
VIP
Neuron Types
E
Spike half-width (ms)

Supplementary Figure 5. A. Experiment workflow of *in vivo* 2-p targeted electrophysiology experiments. Vm was recorded ~2 – 5 minutes after whole-cell configuration was achieved (break-in), followed by mapping of orientation tuning curves with whole-screen sinusoidal drifting gratings (Methods). In a subset of cells, intrinsic membrane properties were measured by current steps, and manipulations including changes of anesthesia depth and/or current injections might be conducted, depending on the quality of the seal. **B.** Example trace of raw (unfiltered) spontaneous Vm recorded (left column) from the Pyr neuron shown in Fig. 3C – E (Pyr#15) before (black) and after (blue) spike removal using median filtering (top) or removing Vm data within -5 – 5 ms around detected spikes (bottom). Insets show the extended views of the Vm data in the same red box. Vm skewness estimated by ξ_M and ξ is denoted in the Vm distribution plots (right column), respectively. **C.** Vm consistency across transgenic mouse lines. Example tdT+ neurons recorded in a *Rorb-IRES-Cre;Ai14* (left, Pyr#4) and *Scnn1a-IRES-Cre;Ai14* (right, Pyr#10) mice under Isoflurane anesthesia showing normal Vm responses (unfiltered) to current steps, which is comparable to the *Cux2-IRES-CreERT2;Ai14* neuron (Pyr#15) shown in Fig. 3C – E. **D.** Depth distribution of whole-cell (black) and cell-attached (gray) recordings in Pyr, SST, PV and VIP neurons. **E.** Population data of spike width in whole-cell (black) and cell-attached (gray) recordings in Pyr, SST, PV and VIP neurons. Note there was no overlap between SST and PV cells.

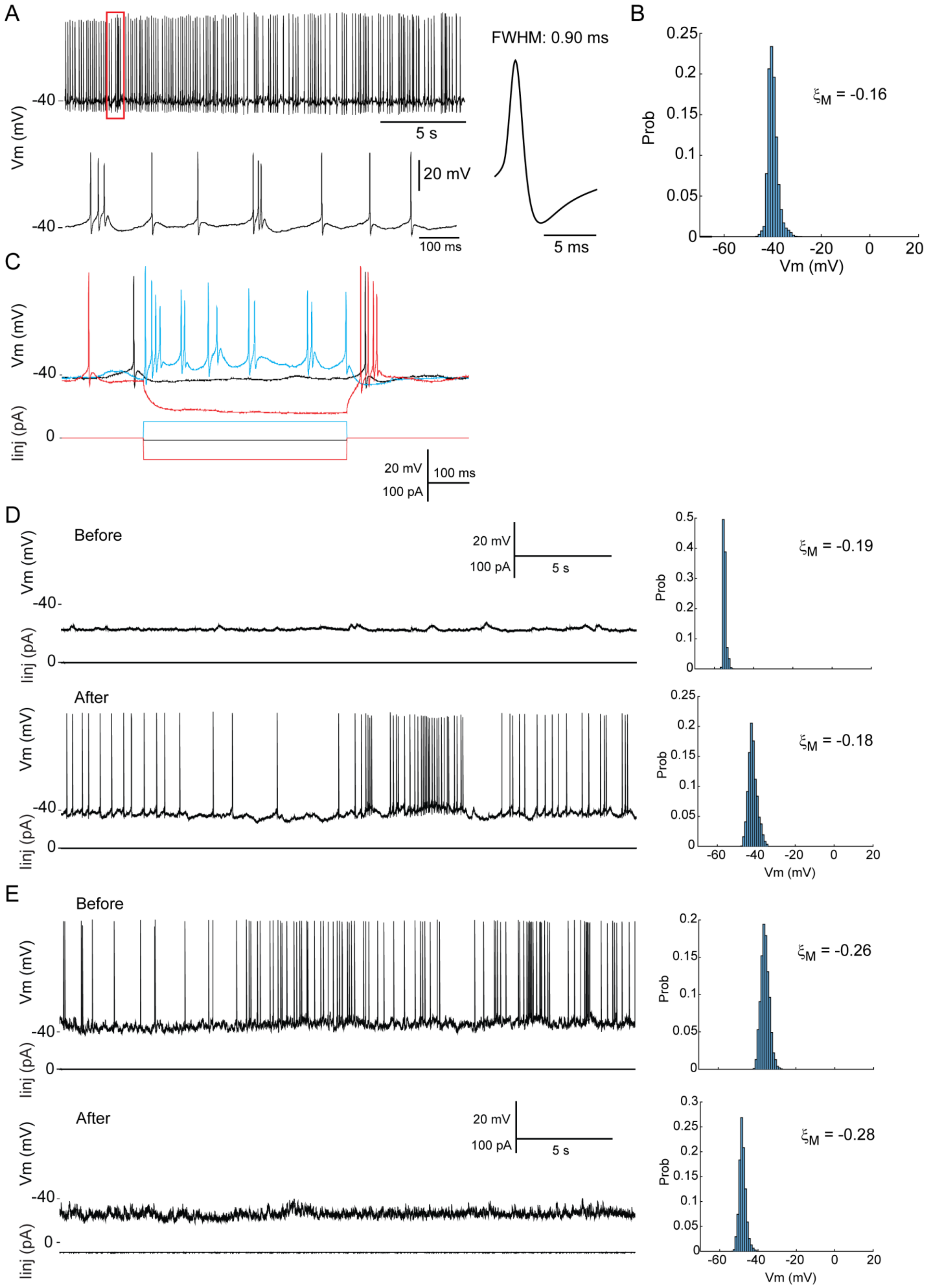

A
Vm (mV)
-40
5 s
20 mV
100 ms
FWHM: 0.90 ms
5 ms
B
Prob
ξ_M = -0.16
Vm (mV)
C
Vm (mV)
Iinj (pA)
20 mV
100 pA
100 ms
D
Before
After
ξ_M = -0.19
ξ_M = -0.18
E
Before
After
ξ_M = -0.26
ξ_M = -0.28
5 s

Supplementary Figure 6. A – C. Consistency of Vm characteristics in SST Subtype I neurons. **A.** Example unfiltered spontaneous Vm trace (left) recorded from an SST Subtype I neuron (SST#2) under urethane anesthesia showing high spontaneous firing (top) and spike adaption, as illustrated by the extended view of data within the red box (bottom). This SST cell was a regular-spiking cell as suggested by its spike width of 0.9 ms (right). **B.** Unimodal Vm distribution of the SST neuron in **A** after spike removal. Note the similarity to Fig. 4C, suggesting the observed soloist behaviour of Subtype I SST neurons is not an anesthesia artifact. **C.** Normal intrinsic membrane properties in response to current steps. Note the absence of synaptic activity even when the Vm was driven away from its reversal potential by hyperpolarizing current steps (also see **E**) compared with Subtype II chorister SST neurons (Fig. 4D and Supplementary Figure 7), suggesting little net excitatory drive. The spike width and the adapting spiking together indicate this neuron is not a fast-spiking PV neuron. **D & E.** The unimoality of Vm distribution in Subtype I SST neurons is independent of Vrest or the depth of anesthesia. **D.** Top: Example raw Vm trace of a spontaneously silent Subtype I SST neuron (SST#13) with a relatively more hyperplorized Vrest under Isoflurane anesthesia. Neurons like this silent SST cell couldn't be detected by extracellular unit recordings. Bottom: Example Vm trace of the same Subtype I SST neuron (SST#13) 15 mins after decreasing Isoflurane concentration. Note that this SST neuron became active but Vm distribution remained unimodal. **E.** Top: Example Vm trace of a spontaneously active Subtype I SST neuron (SST#16) under Isoflurane anesthesia. Note the similarity of Vm to the cell in **A – C**. Bottom: Example Vm trace of the same SST Subtype I neuron (SST#16) with hyperpolarizing current injection. Note that this neuron no longer actively fired action potentials but Vm distribution remained the same. These data suggested Subtype I SST neurons were intrinsically soloist-behaving.

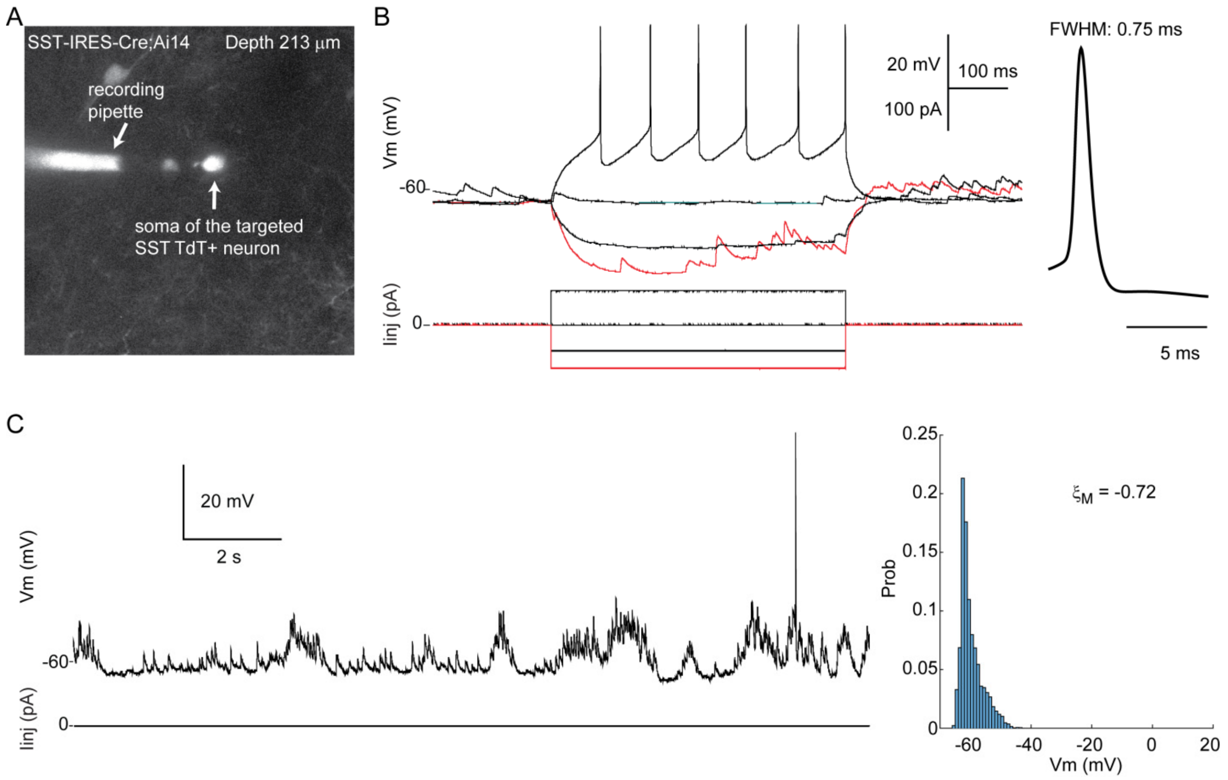

Supplementary Figure 7. A – C. Consistency of Vm activity between SST Subtype II neurons. **A.** Z-projection image of a Subtype II SST neuron (SST#9) after the whole-cell configuration was achieved. The cell was recorded at 213 μm underneath the pia under urethane anesthesia. **B.** Normal intrinsic membrane properties in response to current steps. Note the spontaneous Vm fluctuation when hyperpolarizing currents were injected. This SST cell was a regular-spiking cell, as suggested by its spike width (right). **C.** Example spontaneous Vm trace (left) recorded from SST#9 showing apparent spontaneous Vm oscillation, suggesting a strong net excitatory drive, and consistently, Vm showed a highly skewed distribution (right).

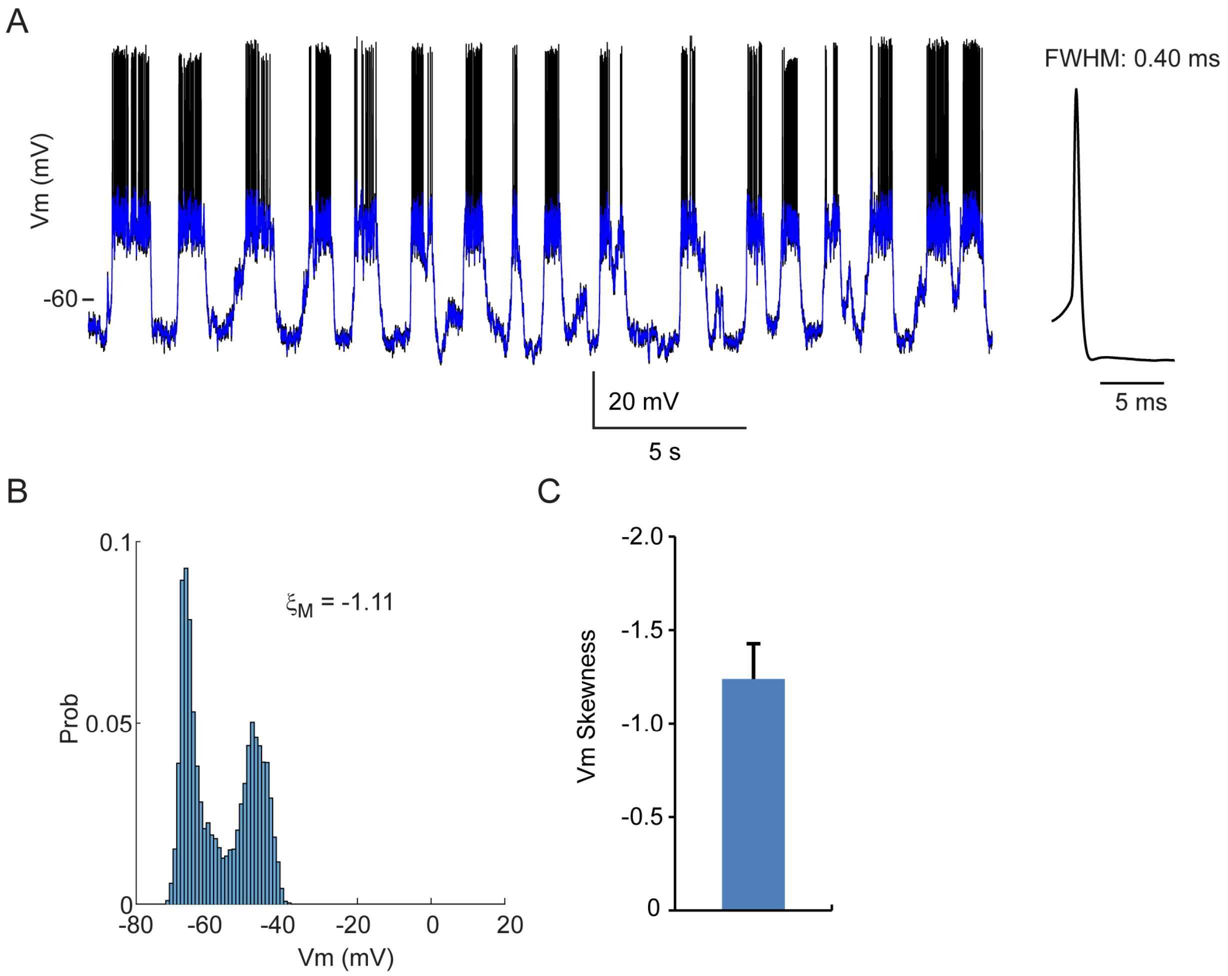

Supplementary Figure 8. PV neurons had bimodal Vm distributions. **A.** Example spontaneous Vm trace (left) recorded from a PV neuron (PV#3) before (black) and after (blue) removing spikes by median filtering. Note that this PV neuron showed apparent spontaneous Vm oscillations, in stark contrast to the unimodal Vm distribution observed in SST Subtype I neurons. Identity of this cell was double-confirmed by the expression of tdT and the narrow (0.4 ms) spike width (right). **B.** Consistently, Vm showed a skewed, bimodal distribution. **C.** Population data of Vm skewness ξ_M in PV neurons (n = 3).

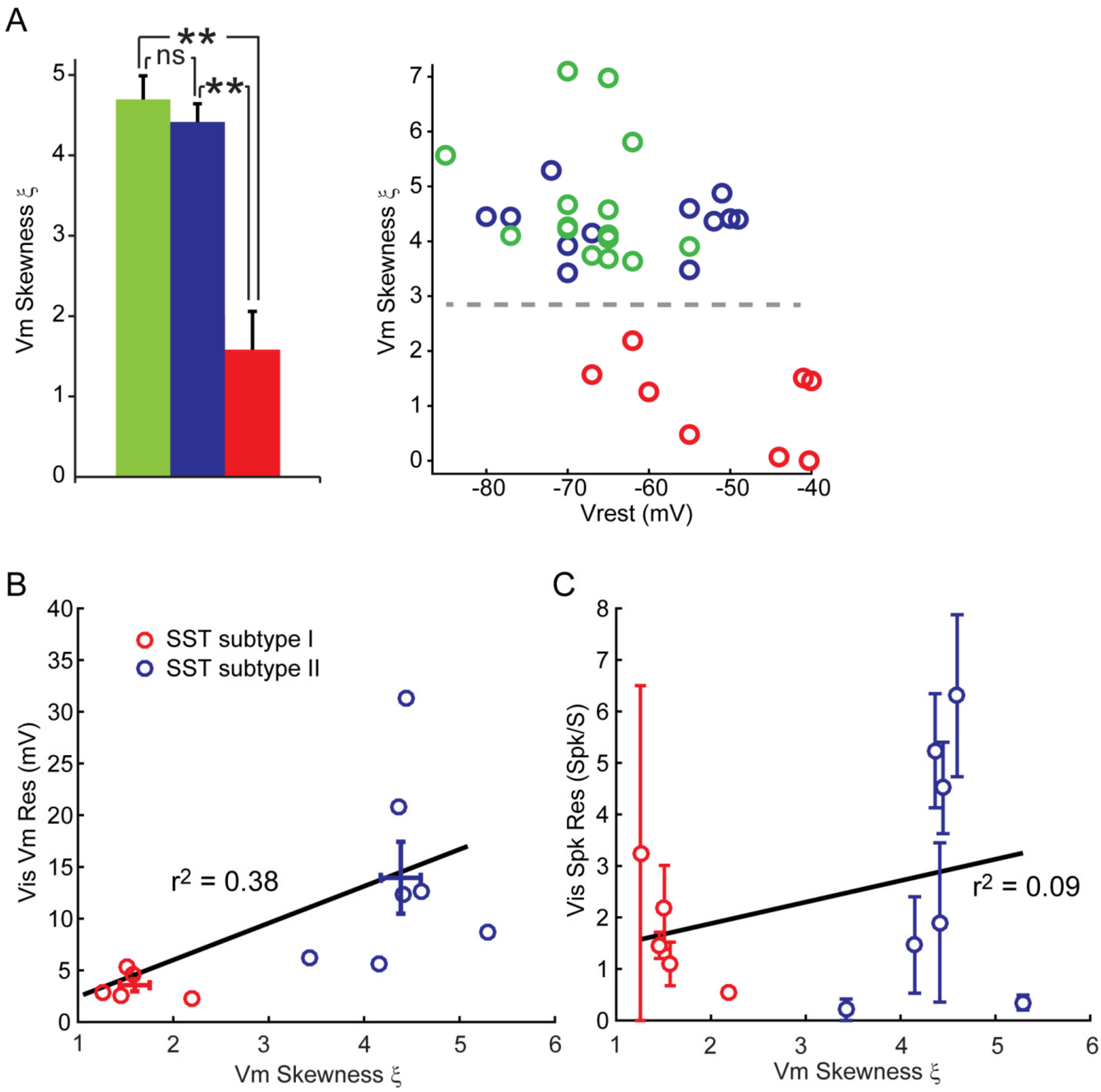
A
**
ns
**
Vm Skewness ξ
5
4
3
2
1
0
7
6
5
4
3
2
1
0
-80
-70
-60
-50
-40
Vrest (mV)
B
C
SST subtype I
SST subtype II
Vis Vm Res (mV)
40
35
30
25
20
15
10
5
0
r2 = 0.38
Vm Skewness ξ
1
2
3
4
5
6
Vis Spk Res (Spk/S)
8
r2 = 0.09

Supplementary Figure 9. Confirmation of SST subtype classification using ξ, an alternative measure of Vm skewness. **A.** Left: Population data of ξ of Pyr, SST Subtype I and II neurons. Right: Pyr, Subtype I and II SST INs can be separated by ξ and Vrest, consistent with Fig. 4H. **B & C**. Vm data are more informative than spiking. **B.** Vm deflection magnitude plotted against Vm skewness ξ. Subtype I and II SST INs can be clearly separated, consistent with Fig. 5G. **C**. No apparent dependence of visually evoked spiking response magnitude on Vm skewness ξ.

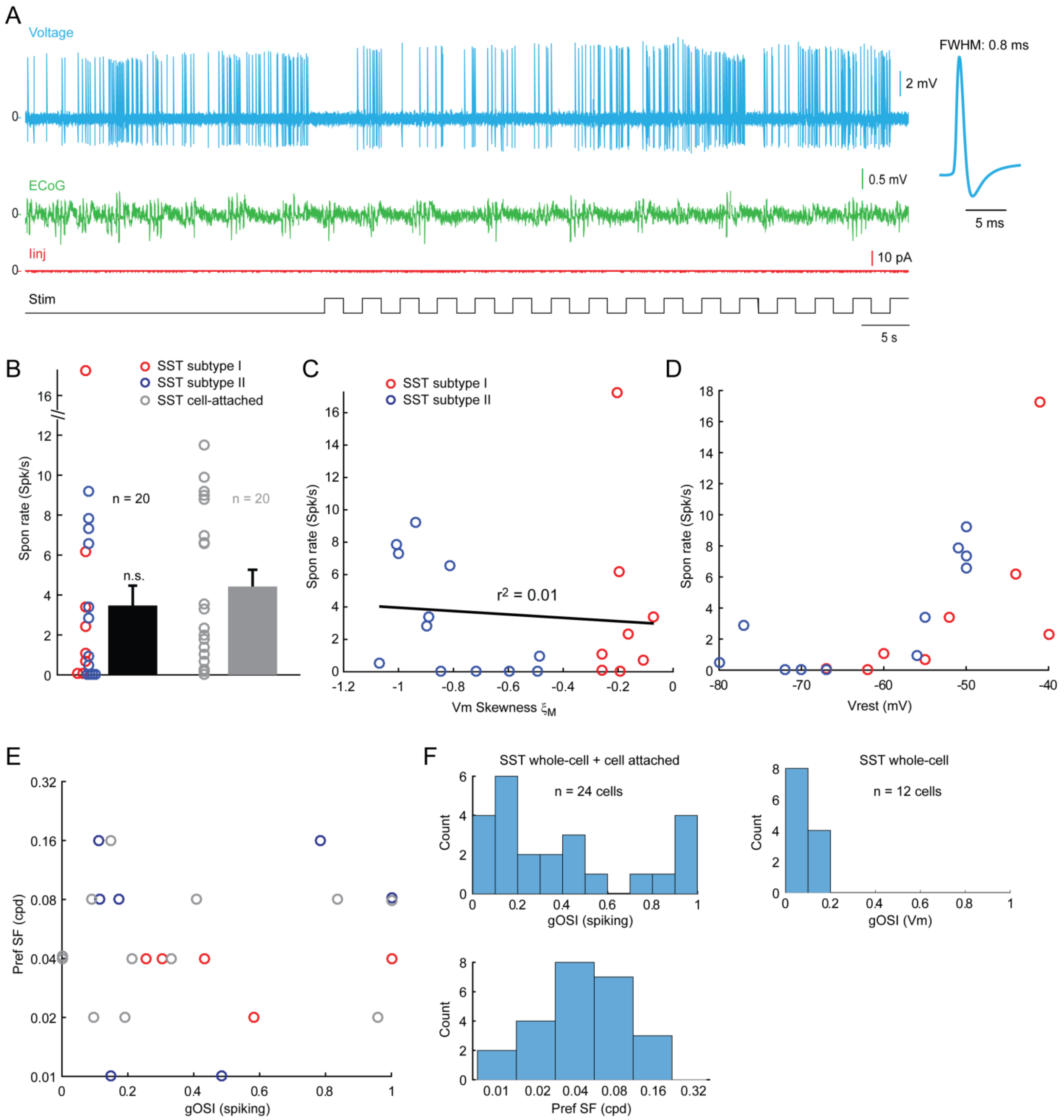
A
Voltage
ECoG
Iinj
Stim
2 mV
0.5 mV
10 pA
5 s
FWHM: 0.8 ms
5 ms
B
SST subtype I
SST subtype II
SST cell-attached
Spon rate (Spk/s)
n = 20
n.s.
n = 20
C
SST subtype I
SST subtype II
Spon rate (Spk/s)
r2 = 0.01
Vm Skewness ξM
D
Spon rate (Spk/s)
Vrest (mV)
E
Pref SF (cpd)
gOSI (spiking)
F
SST whole-cell + cell attached
n = 24 cells
Count
gOSI (spiking)
SST whole-cell
n = 12 cells
Count
gOSI (Vm)
Count
Pref SF (cpd)

Supplementary Figure 10. Visually evoked responses in SST neurons. A. Example cell-attached recording of an SST neuron 200 μm below the pia surface showing the spiking activity (blue) and ECoG (green) under Isoflurane anesthesia. This neuron was regular-spiking, and the average shape of its spikes was shown on the right. **B**. Spontaneous firing rate of SST neurons from whole-cell (n = 20) and cell-attached (n = 20) recordings. Whole-cell recording has a higher chance (30%, 6 of 20) of sampling inactive cells than cell-attached recordings (5%, 1 of 20). **C** & **D.** Spontaneous firing rate was not in correlation with Vm Skewness ξ_M, but nearly exponentially increase with Vrest in SST neurons. **E.** Population data of the global Orientation Selectivity Index (gOSI) and preferred spatial frequency (pref SF) of SST neurons (n = 24) acquired by whole-cell (n = 12) and cell-attached recordings (n = 12). gOSI was calculated from spiking responses. **F.** Left column: Histograms of gOSI (top) and pref SF (bottom) of SST cells shown in **E**. These distributions were consistent with previous findings. Right column: Histogram of gOSI measured by Vm response deflections (gOSIVm).
